# Carnauba Wax Coatings Enriched with Essential Oils or Fruit By-Products Reduce Decay and Preserve Postharvest Quality in Organic Citrus

**DOI:** 10.3390/foods14152616

**Published:** 2025-07-25

**Authors:** Lorena Martínez-Zamora, Rosa Zapata, Marina Cano-Lamadrid, Francisco Artés-Hernández

**Affiliations:** 1 Postharvest and Refrigeration Group, Department of Agricultural Engineering and Institute of Plant Biotechnology, Universidad Politécnica de Cartagena, 30203 Cartagena, Murcia, Spain; rosa.zapata@upct.es (R.Z.); marina.canol@umh.es (M.C.-L.); fr.artes-hdez@upct.es (F.A.-H.); 2 Department of Food Technology, Nutrition, and Food Science, Faculty of Veterinary Sciences, University of Murcia, 30071 Espinardo, Murcia, Spain

**Keywords:** *Citrus limon*, *Citrus sinensis*, valorization, phenolic compounds, in vitro, in vivo, natural antifungals, *Penicillium digitatum*, carnauba wax formulations

## Abstract

This research analyzes the innovative development of carnauba wax coatings enriched with essential oils (EOs: lemon, orange, grapefruit, clove, oregano, and cinnamon) or fruit by-products (FBPs: avocado, tomato, carrot, orange, lemon, and grapefruit) to improve postharvest preservation of organic oranges and lemons. Six EOs and six FBPs were evaluated for total phenolic content (TPC) and in vitro antifungal activity against *Penicillium digitatum*. Based on results, grapefruit, oregano, and clove EOs were selected for lemons, while avocado, orange, and grapefruit FBPs were selected for oranges. An in vivo test at 20 °C for 15 days with carnauba wax coatings assessed antifungal performance. Clove EO and avocado FBP showed strong in vitro inhibition and consistent hyphal suppression (~100 and ~82%, respectively). In vivo, coatings with grapefruit EO and avocado FBP significantly reduced fungal decay and sporulation (~75%) in lemons and oranges, respectively. Coated fruits also retained weight losses by ~25% compared to uncoated ones. These findings suggest that phenolic-rich natural extracts, especially from agro-industrial residues like avocado peels, offer a promising and sustainable strategy for postharvest citrus disease control. Further studies should test coating effectiveness in large-scale trials under refrigeration combined with other preservation strategies.

## 1. Introduction

Citrus fruits represent one of the most extensively cultivated crop groups worldwide, with their global agricultural footprint reaching millions of hectares annually. According to the FAO, mandarin-related cultivars, oranges, lemons and limes, and grapefruits are grown on approximately 3.9, 3.4, 1.4, and 0.4 M ha, respectively [1]. Major producers include Brazil, China, the United States, Mexico, India, and Spain. Within this context, oranges and lemons stand out not only for their economic relevance but also for their high postharvest vulnerability, particularly to fungal infections that compromise fruit quality and marketability.

Citrus fruits, being non-climacteric, do not continue ripening after harvest. This characteristic makes them ideal candidates for postharvest treatments such as wax-based coatings, which serve to reduce moisture loss, slow down respiration, and offer a physical barrier against microbial invasion [2]. These coatings are typically water- or alkali-based emulsions containing natural or synthetic waxes (e.g., esters, resins), along with stabilizers like plasticizers or antifoaming agents. Among the methods employed for their application—immersion and brushing—both aim to achieve uniform coverage and optimal coating thickness [3]. The widespread use of these techniques has significantly advanced the preservation technology for citrus, extending shelf life and minimizing spoilage during storage and transport.

In recent years, regulatory and environmental pressures have driven a shift towards more sustainable and organic-compatible postharvest treatments. For citrus designated as organic in the European Union, coatings must be biodegradable, composed of natural ingredients, and safe for human consumption. Carnauba wax, derived from the leaves of *Copernicia cerifera*, has emerged as a leading candidate due to its glossy finish and high content of long-chain fatty acids such as cerotic, lignoceric, montanic, and behenic acids [4,5].

Despite these advancements, the citrus industry continues to face postharvest losses primarily due to *Penicillium digitatum*, the causative agent of green mold, which is responsible for up to 90% of decay in stored citrus fruit [6]. Current synthetic fungicide-based strategies, although effective, raise concerns about resistance development, environmental impact, and consumer health [3]. Consequently, research has increasingly focused on the development of natural alternatives with antifungal properties. In this context, coatings rich in bioactive compounds derived from fruit and vegetable by-products (FBPs) have gained significant attention.

The global agri-food sector generates a considerable amount of waste, with fruit and vegetable processing alone accounting for up to 30% of the total yield [7]. These by-products, often discarded or relegated to low-value uses such as animal feed or biomass, are rich sources of high-value bioactive compounds with important techno-functional abilities as antioxidant, antimicrobial, and antifungal agents [8,9]. Furthermore, the obtention of essential oils (EOs) from these discards has also gained attention due to their antimicrobial and antifungal potential [10]. For instance, compounds as thymol, carvacrol, and citral from EOs or polyphenols, flavonoids, and organic acids from FBPs are known to disrupt microbial membranes, interfere with enzyme activity, and inhibit cell proliferation, which supports their potential use in food preservation as antimicrobials and antifungals [9,10,11,12].

The integration of these bioactive-rich extracts into wax-based coatings offers a novel approach to enhance the protective functions of commercial waxes like carnauba [12]. Such fortified coatings could serve dual purposes: preserving fruit quality through physical means and providing biochemical defense against postharvest pathogens such as *P. digitatum*. The optimization of these coatings entails the selection of appropriate by-product sources, development of eco-friendly extraction methods, and rigorous in vitro and in vivo evaluation to assess antifungal efficacy and overall performance in real-world storage conditions. As its main novelty, this study presents a pioneering side-by-side comparison between EOs and FBPs under identical conditions. Moreover, it introduces a novel combination of avocado peel FBP and carnauba wax as an antifungal coating for citrus, which outperforms traditional EO-based coatings in terms of sensory neutrality.

This study aims to explore the potential of bioactive coatings formulated from FBPs and EOs, focusing on their application to organically grown citrus fruits, specifically lemon and oranges. By integrating six EOs and phenolic-rich extracts from six FBPs into carnauba-based wax emulsions, we evaluated their effectiveness against *P. digitatum* through comprehensive in vitro and in vivo trials. This research contributes to the sustainable valorization of agri-food waste and the development of greener, safer alternatives for citrus postharvest protection.

## 2. Materials and Methods

### 2.1. Obtention of Essential Oils and Fruit By-Products and Initial Characterization

A screening of the in vitro and in vivo antifungal activity of 6 EOs and 6 FBPs was evaluated. EOs supplied by AromaLabs (Lyon, France) were obtained from citrus and herbs. Citrus EOs were from lemon (*Citrus limon* L.), orange (*Citrus sinensis* L.), and grapefruit (*Citrus paradisi*), whose principal components were limonene, linalool, citral, and geraniol. Herbs EOs were from clove (*Eugenia carophyllus*: eugenol and isoeugenol), oregano (*Origanum vulgare* L.: limonene, linalool, carvone, citral, eugenol, and geraniol), and cinnamon (*Cinnamomum aromaticum* L.: limonene, eugenol, benzaldehyde, alcohol, and cinnamaldehydes).

FBPs were obtained from avocado peels, industrial tomato by-products, carrot pomace, orange peels, lemon peels, and grapefruit peels. ‘Hass’ avocados (Trops SAT 2803, Málaga, Spain) were used to obtain peels as previously described [13]. Industrial tomato by-products from Bonnysa Group (Alicante, Spain) were obtained as described [14]. Carrot pomace was obtained from the juice of non-commercialized carrots from Hortalizas Requena S.L. (Murcia, Spain) as described [15]. Lemon, orange, and grapefruit peels were manually obtained from non-commercialized organic fruits from Toñifruit S.L. (Librilla, Murcia, Spain) as described [16].

The total phenolic content (TPC) of the FBPs and EOs was quantified using UV–visible spectrophotometry with a microplate reader (Infinite M200, Tecan, Männedorf, Switzerland). TPC was determined through the Folin–Ciocalteu protocol [17]. Samples of 19 μL of methanolic extracts, obtained as previously described [16], were placed into 96-well flat-bottom polystyrene plates (Greiner Bio-One, Frickenhausen, Germany), followed by 29 μL of 1 N Folin–Ciocalteu reagent. After a 3 min reaction time in darkness, 192 μL of a sodium carbonate (0.4%) and sodium hydroxide (2%) aqueous solution was added to initiate the reaction. The microplates were incubated for 1 h in darkness, and absorbance was read at 750 nm. Gallic acid was used as a reference standard, and TPC values were reported as g of gallic acid equivalents (GAE) per kg; y = 4.4497x + 0.0303; R^2^ = 0.9957.

### 2.2. Plant Material and Initial Characterization

Organic lemons (*Citrus limon* cv. Verna) and oranges (*Citrus sinensis* cv. Navelate) were harvested in May 2023 and May 2024, respectively. After harvesting, washing (2% peroxiacetic acid with Citrocide PC, Citrosol, Valencia, Spain) and handling at Toñifruit S.L. (Librilla, Murcia, Spain), fruits were transported (~50 min) to the facilities of Universidad Politécnica de Cartagena (Cartagena, Spain).

The physicochemical quality attributes were initially measured, and throughout a shelf-life of 15 days at room temperature (20 ± 3 °C) following the application of the corresponding treatment (*n* = 20). Each fruit’s weight was determined using a precision balance (Gram Precision, S.L., Barcelona, Spain) to measure the percentage of dehydration along the shelf-life. The equatorial and longitudinal diameters as well as the peel thickness were measured with a digital caliper (Mitutoyo; Neuss, Germany). Firmness was determined at room temperature to simulate normal handling conditions using a universal testing machine (Ibertest S.A.E., Madrid, Spain), measuring the displacement (mm) of an 8 cm diameter probe when applying a constant compression force of 15 N at a speed of 2 mm/s on the equatorial area of the fruit. Results were expressed as the percentage of deformation, calculated as the ratio between the displacement (mm) and the equatorial diameter (mm) of the fruit. The external color of the fruits was measured in the CIELab system using a Konica Minolta CR-400 colorimeter (Tokyo, Kanto, Japan). Juice yield was also determined, and, from these juices, pH was measured with a digital pH meter (GLP21, Crison; Alella, Catalonia, Spain), and total soluble solids (TSSs) were measured with a portable digital refractometer (Atago N1; Tokyo, Kanto, Japan).

### 2.3. In Vitro Antifungal Activity Against Penicillium digitatum

The in vitro antifungal activity of 1% and 5% FPBs and 0.1%, 0.5%, 1%, and 2% EOs was assessed to determine their effectiveness against the development of *P. digitatum* using potato dextrose agar (PDA) enriched in these FPBs and EOs according to previous literature [18]. Tween 80 at 1% was used as an emulsifier. For that, under aseptic conditions within a laminar flow cabinet (SpainTelstar BIO-I-A/M; Barcelona, Spain), the sterilized mixtures were homogeneously distributed into sterile Petri dishes. After the medium solidified, 10 μL of a *P. digitatum* spore suspension (1 × 10^5^ CFU/mL) was added. Each treatment was incubated for 7 d at 25 °C in a controlled environment chamber (Trade Raypa, Barcelona, Spain). Daily measurements of fungal hyphae and spore growth were taken (mm) using the Image J software v1.54p (Wayne Rasband, Kensington, MD, USA) to determine the diameter growth and the corresponding inhibition capacity of each treatment (*n* = 5).

### 2.4. In Vivo Antifungal Activity Against Penicillium digitatum

**Figure 1:**
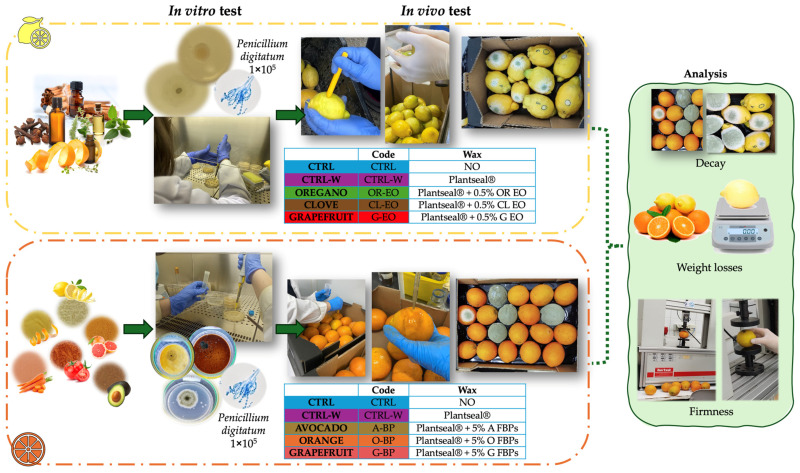
Experimental design.

Twenty fruits in tray boxes were artificially inoculated at the equatorial zone using 2 mm sterile punches dipped in a *P. digitatum* spore suspension (1 × 10^5^ CFU/mL), as previously described [19]. Five boxes per treatment were inoculated (*n* = 100). Coatings were applied after 15 h from the inoculation to assess the curative antifungal potential of each treatment. The in vivo antifungal efficacy of the treatments was performed using Plantseal^®^ carnauba wax-based coatings (Productos Citrosol S.A., Valencia, Spain) enriched with 5% FBP (avocado, orange, and grapefruit peels) or 0.5% EO (oregano, clove, and grapefruit EOs), which were selected based on their in vitro TPC and antifungal activity. In addition, uncoated fruits were used as a negative control (CTRL), and a commercial wax coating (Plantseal^®^) without any supplementation was used as a coating control (CTRL-W). Wax formulations were prepared by heating the commercial carnauba wax to 45 °C in a water bath, followed by the addition of the selected 5% FBP or 0.5% EO. Treatment abbreviations and compositions are detailed in Figure 1. The mixtures were homogenized by vigorous agitation in an orbital shaker for 10 min at 200 rpm. After this, enriched carnauba waxes were individually applied using 1 mL per fruit, homogenizing on the surface with the hand, and dried with warm air for a few minutes to avoid wax deposits on the surface of the fruit, as previously described [20]. Daily monitoring of artificial decay on these fruits was performed by counting to determine the antifungal potential of the studied coatings.

### 2.5. Shelf-Life Study

Citrus fruits were randomly divided into treatment groups and coated as described above. A total of boxes containing 20 oranges and 10 boxes containing 10 lemons (*n* = 100; 5 repetitions of 20 replicates for oranges and 10 repetitions of 10 replicates for lemons), arranged in protective trays, were used to measure the in vivo antifungal activity against *P. digitatum* and 6 boxes with 15 fruits each were prepared to perform the shelf-life study with 2 sampling days, after 7 and 15 d (*n* = 45 per sampling day, having 5 repetitions of 5 replicates per fruit) at room temperature (20 ± 3 °C) to simulate accelerated shelf-life conditions, totalling 950 fruits (~160 kg lemons and ~300 kg oranges). Coatings were applied manually as described above. Quality attributes, including weight loss, firmness, external color, and natural decay, were assessed as described in the above sections.

### 2.6. Statistical Analysis

The experiment assessed the significance of differences in the in vitro TPC of FPBs and EOs, as well as their in vitro antifungal activity against *P. digitatum* and the in vivo physicochemical quality parameters of wax-coated citrus. To analyze the results, one-way and two-way analysis of variance (ANOVA) tests were performed (*p* < 0.05) using Statgraphics Centurion software (v. XV.II, Statgraphics Technologies Inc.; The Plains, VA, USA). Differences between group means for in vitro inhibition results were compared and evaluated for significance using the Least Significant Difference (LSD) multiple range test to evaluate the interaction among the studied variables: day of growth, type of FBP or EO, and dose (%).

## 3. Results

### 3.1. Initial Characterization of Fruits and Ingredients

Initial results of characterization of the studied citrus are presented in Table 1, in which it can be appreciated that both lemon and oranges reached the optimum quality to be harvested once they were taken to develop the present experiments.

Table 1 summarizes the initial quality parameters of lemons and oranges used in the study, including weight, diameter, peel thickness, firmness, color (L*, a*, b*), juice yield, pH, and total soluble solids (TSSs) content. These measurements provide a baseline for evaluating postharvest changes and coating efficacy. The results confirm expected varietal differences. For instance, lemons exhibited higher acidity and lower juice yield compared to oranges. As well as the thickness of lemon skin was higher than in oranges, as this work was developed with the Verna cv. Also, the initial visual quality of the studied fruits after washing can be appreciated in this Table 1.

**Figure 2:**
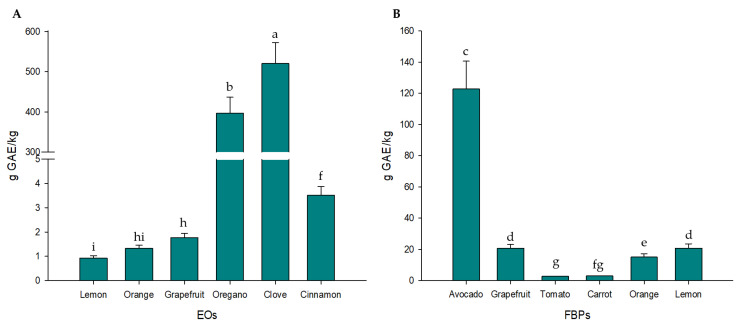
Total phenolic content (g GAE/kg) of EOs (**A**) and FBPs (**B**) studied in the present work. Different letters represent significant differences (*p* < 0.05) among EOs and FBPs.

TPC of EOs and FBPs tested is shown in Figure 2A,B, respectively. The statistical differences denoted by different letters demonstrate significant variability among samples (*p* < 0.05), both EOs and FBPs. Among EOs, oregano exhibited the highest TPC, whereas avocado peel extract showed the highest value among FBPs. In this sense, the compounds with the highest TPC were oregano EO, clove EO, and avocado FBP, followed by citrus (grapefruit, lemon, and orange) FBPs, cinnamon EO, tomato and carrot FBPs, and citrus (grapefruit, lemon, and orange) EO. These results highlight the potential of these natural sources as functional ingredients with antioxidant and antifungal properties. The selection of ingredients for coating formulation was guided by these results, prioritizing those with the highest phenolic concentrations to perform the in vivo test.

### 3.2. Results of In Vitro Antifungal Activity Against Penicillium digitatum

**Figure 3:**
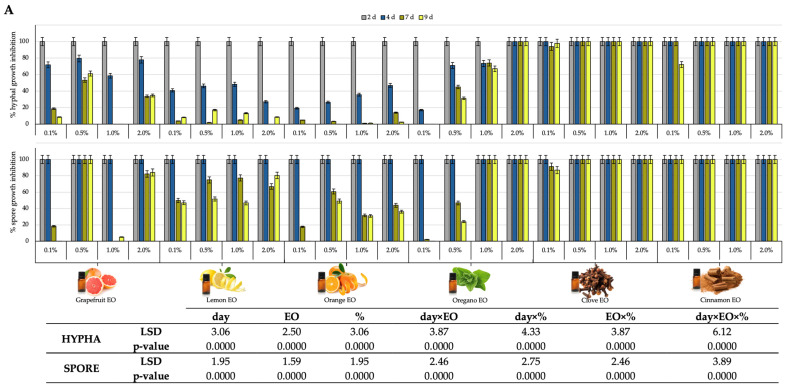

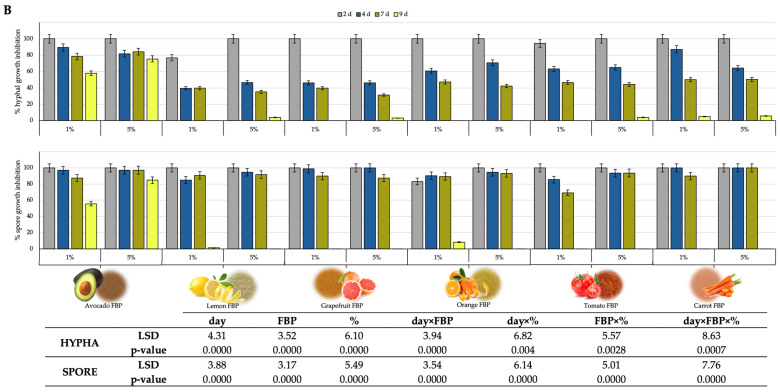
In vitro hyphal and spore growth inhibition of *P. digitatum* compared to control plates of tested EOs ((**A**): grapefruit; lemon; orange; oregano; clove; cinnamon) and FBPs ((**B**): avocado; lemon; grapefruit; orange; tomato; carrot).

Figure 3 shows the inhibitory effects of EOs (A) and FBPs (B) on the mycelial growth of *P. digitatum*, the main postharvest pathogen in citrus fruits, compared to control plates. The hyphal diameter of control plates of *P. digitatum* after 2, 4, 7, and 9 d was 32 ± 4.5, 45 ± 2.6, 63 ± 3.9, and 75 ± 5.8 mm, while spore diameter for these days was 25 ± 2.7, 33 ± 1.7, 47 ± 1.9, and 55 ± 6.8 mm, respectively.

As shown, fungal growth in most of the plates enriched in EOs and FBPs started after 4 d of incubation, except for 1% lemon FBP, which showed a 78 ± 4% reduction in hyphal growth after 2 days of incubation. In this sense, all the EOs and FBPs studied showed high inhibition abilities of the spore growth of *P. digitatum* after 2 and 4 d incubation, with the EOs being the most antifungal ingredients against this fungus. Specifically, clove and cinnamon EOs reported the highest inhibition rates for the growth of this fungus, although these two compounds reported that strong odors were directly incorporated into the coating. In this sense, oregano, orange, and grapefruit were chosen due to their high ability against the spore growth at low concentrations (0.5%). Regarding the antifungal ability of FBPs, all the studied extracts showed a high inhibition against the spore growth, although only avocado FBP showed a high inhibition of hyphal growth during all the incubation time, reaching values of 75 ± 4% inhibition after 9 d incubation at 5% concentration. These results correlate well with the TPC presented in Figure 2 and support the hypothesis that phenolic-rich natural extracts exert strong antifungal effects. The findings provide a scientific basis for the selection of these compounds in the formulation of the coatings tested in subsequent in vivo experiments. Furthermore, the dried and milled FBPs that were tested did not release intense odors as EOs did, which can also justify their use in coatings.

### 3.3. Results of In Vivo Antifungal Activity Against Penicillium digitatum

**Figure 4:**
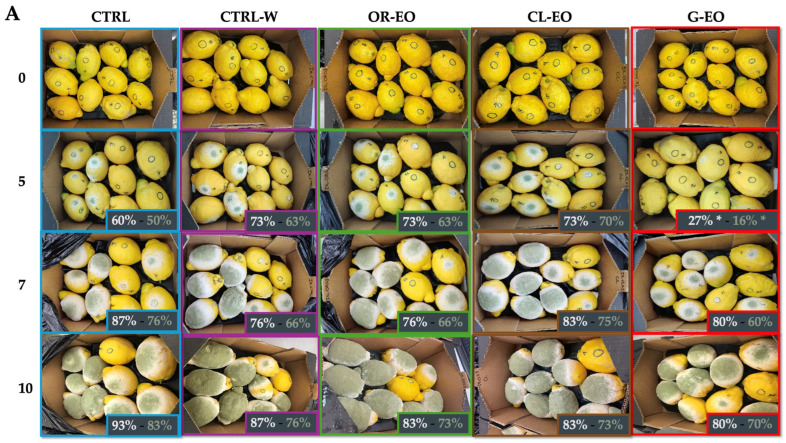

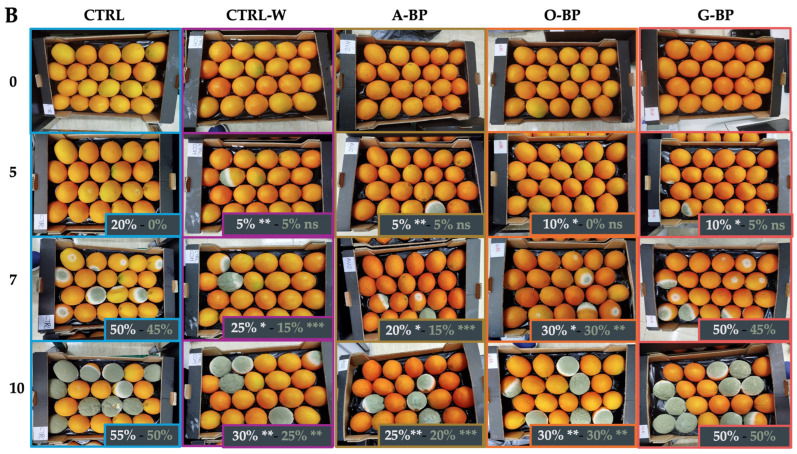
Decay (%) and sporulation (%) percentage of inoculated organic lemon and oranges with *P. digitatum* and coated with waxes enriched in EOs (**A**) and FBPs (**B**). CTRL: control unwaxed; CTRL-W: control waxed; OR-EO: wax enriched in 0.5% oregano EO; CL-EO: wax enriched in 0.5% clove EO; G-EO: wax enriched in 0.5% grapefruit EO; A-BP: wax enriched in 5% avocado FBP; O-BP: wax enriched in 5% orange FBP; G-BP: wax enriched in 5% grapefruit FBP. ns: no significant results compared to CTRL; *, **, and *** denotes significant results *p* < 0.05, *p* < 0.005, and *p* < 0.001, respectively, compared to CTRL. Bar charts of these data are shown in Figure S1.

Figure 4 shows the visual growth of *P. digitatum* during 10 d at 20 ± 3 °C in the in vivo experiments with organic lemons (A) and oranges (B) coated with carnauba wax enriched in EOs and FBPs, respectively. Figure S1 shows the bar charts with the mean and standard deviation of the obtained values.

As shown, the application of carnauba wax-based coatings, especially those enriched with grapefruit EO (G-EO) and avocado peel extract (A-BP), for the studied organic lemons and oranges, respectively, significantly reduced both the incidence of infection and the level of sporulation compared to uncoated CTRL. Separately, in the case of lemons, we can observe a high incidence of the *P. digitatum*, which affected most of the half of the studied lemons after 5 days of incubation, except for G-EO, which showed a delay in the decay percentage of 2 days in comparison with the rest of the EO-coated and uncoated (CTRL) lemons.

For organic oranges, the fungus development was more controlled. In fact, all coated treatments showed important reductions in the decay and sporulation incidence compared to the uncoated treatment (CTRL). However, A-BP showed the most promising results with a reduction after 10 d incubation at 20 °C of ~30 ± 2% compared to CTRL and of ~5 ± 0.4% compared to CTRL-W, which also showed a good reduction in the decay and sporulation index, with no differences with O-BP. Nevertheless, G-BP did not show any improvement in the protection against *P. digitatum* growth regarding CTRL. These results confirm the in vivo efficacy of the coatings and suggest a synergistic effect between carnauba wax and the added bioactive compounds, especially from avocado peels. These findings demonstrate that the antifungal activity observed in vitro is maintained or even enhanced under real storage conditions.

**Figure 5:**
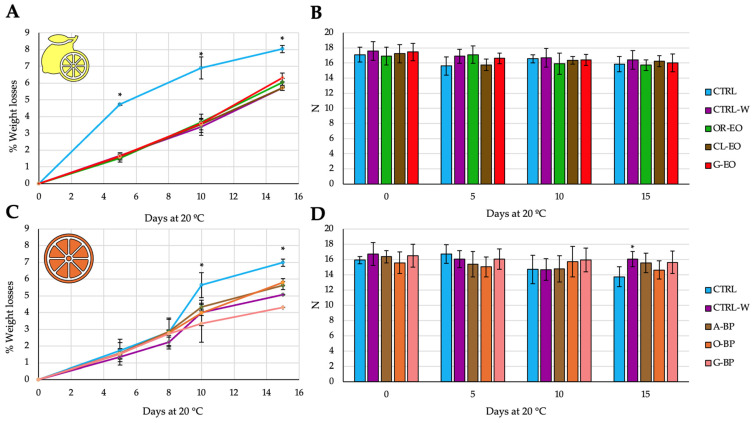
Weight losses due to dehydration (%) and firmness (N) of organic lemon ((**A**): weight losses; (**B**): firmness) and oranges ((**C**): weight losses; (**D**): firmness) coated with waxes enriched in EOs and FBPs, respectively. CTRL: control unwaxed; CTRL-W: control waxed; OR-EO: wax enriched in 0.5% oregano EO; CL-EO: wax enriched in 0.5% clove EO; G-EO: wax enriched in 0.5% grapefruit EO; A-BP: wax enriched in 5% avocado FBP; O-BP: wax enriched in 5% orange FBP; G-BP: wax enriched in 5% grapefruit FBP. In the case of weight losses, * denotes significant results *p* < 0.05 of CTRL compared to the rest of treatments (no significant differences were found among coated samples), while in the case of firmness, * denotes significant results *p* < 0.05 compared to CTRL.

As a complement to the shelf-life study, Figure 5 presents the evolution of weight loss and firmness in coated and uncoated citrus during storage. Avoiding weight loss and firmness preservation is essential for extending shelf-life and maintaining the sensory quality of citrus fruits during commercial storage and distribution. Coated fruits, particularly those treated with bioactive-enriched wax coatings, exhibited lower weight loss retention over time in lemons and oranges. These effects are attributable to the semi-permeable nature of the coatings, which reduce transpiration and slow down metabolic degradation processes. Nevertheless, the firmness was not affected during the shelf-life study, which could be due to the fact that the short period was monitored. In fact, only a slight tendency of firmness reduction was shown in CTRL oranges at the end of shelf-life compared to coated treatments.

## 4. Discussion

The initial characterization of the studied citrus fruits (Table 1) provides essential reference data for evaluating subsequent effects of coatings. Parameters such as fruit weight, diameter, firmness, juice yield, pH, and TSS confirm varietal differences between Verna lemons and Navelate oranges. In fact, lemons showed greater acidity and thicker peels than oranges, aligning with previous reports as obtained data by González-Molina et al. [21] or Badiche-El et al. [22], who reported similar firmness (13–16 N), juice yield (23.3–29.3%), pH (2.25–2.41), and TSS (6.04–7.48%) for the same variety. Oranges also showed comparable results to previous reports [23,24], who reported TSSs of 11.7–12.6%, a pH of 3.5, or a juice yield of 42% in the same variety. In this sense, the variations in these parameters can influence coating adherence and gas exchange dynamics.

The TPC of studied EOs and FBPs (Figure 2) widely varied with differences among the tested supplements, which is mainly due to the richness of bioactive compounds and the varied origin and nature of every EO or FBP. Regarding EOs, herbs and species showed the highest TPC, with clove and oregano being the most powerful ingredients. These results were expected due to the richness in eugenol from clove [25] and carvacrol from oregano [26,27], both monoterpenoid phenols, and responsible for their characteristic aroma. After these EOs, the richest ingredient in TPC was the FBP from avocado peels [28,29], with ~120 g GAE/kg, and mainly rich in procyanidins and epicatechins with important antifungal, antimicrobial, and antioxidant abilities. Particularly, and because they come from the same fruit family, grapefruit, orange, and lemon FBPs showed similar TPC, being ~20-fold richer in TPC than the EOs obtained from the same fruits. This can be mainly attributed to the high flavonoid content present in the citrus flavedo and albedo [16], both included in the FBPs. In contrast, the EOs were primarily obtained through distillation to capture the aromatic compounds of the flavedo, which is mainly rich in limonene [30], a terpenoid of non-phenolic origin. Lastly, the EO with the lowest TPC was cinnamon, rich in cinnamaldehyde, responsible for its aroma, with known properties as an insecticide, antifungal, and antimicrobial. However, it is not rich in phenolics, as the only phenolics found in its formulation are eugenol and minor compounds as procyanidins [31]. The lowest amount of TPC was found in tomato and carrot pomace, which was also expected due to their richness in carotenoids, such as lycopene or β-carotene, but not in phenolics [32,33]. These results, and the direct correlation between phenolic levels and antimicrobial and antifungal efficacy, support the criteria used for selecting ingredients for in vivo assays.

The in vitro results from this study clearly demonstrated the high antifungal potential of both EOs and FBPs extracts against *P. digitatum*, a major postharvest pathogen in citrus (Figure 3). The inhibitory effects observed, especially on mycelial and spore development, are consistent with previous reports on the bioactivity of plant-derived compounds. The antifungal efficacy of oregano, clove, and grapefruit EOs aligns with findings from multiple studies indicating that volatile oils and phenolic-rich residues can act as potent inhibitors of fungal growth through various mechanisms [34,35].

EOs are well-known for their antimicrobial, antioxidant, and antifungal properties, attributed to their rich content in secondary metabolites such as terpenoids, flavonoids, and phenolic compounds. The pronounced efficacy of clove and oregano EOs in our study reflects their high eugenol and carvacrol content, respectively, both of which are known to disrupt fungal cell walls and inhibit spore germination. This has been previously supported by in vitro research showing strong inhibition of *P. digitatum* and *P. italicum* by clove, thyme, and citrus-derived EOs [6,36]. For instance, Gutiérrez-Pozo et al. [37] showed that 100 ppm of preharvest application of carvacrol was enough to reduce the incidence of the natural growth of this fungus during the refrigerated storage of Fino lemons for 35 d at 10 °C. However, it is important to highlight the limitations associated with EO use, particularly the intense aroma and volatility that may affect consumer acceptability when applied to fresh produce. This sensory issue has been widely reported [34,35,38,39], and our findings reinforce the need to balance antimicrobial effectiveness with odor neutrality. In this context, considering the antifungal ability, the TPC, and the sensory perception, as well as the release rate, clove, oregano, and grapefruit EOs were chosen for the in vivo antifungal test in organic lemons.

In this context, the excellent antifungal performance of avocado peel extract becomes highly relevant. As a FBP from processing, avocado peel contains high concentrations of phenolic acids and flavonoids but lacks the overpowering aroma of EOs, making it an attractive alternative for coating formulations. Our results showed that the avocado FBP extract maintained over 75 ± 4% inhibition of *P. digitatum* hyphal growth after 9 d, a notable performance for a dried and powdered plant material. This sustained antifungal action may be linked to the complex matrix of phenolic compounds, which can persist longer on agar surfaces than volatile EOs [38]. Comparable findings have been reported in the recent literature with plant extracts such as pomegranate peel, olive leaf, and propolis, which exhibited strong in vitro antifungal activity and antioxidant capacity when incorporated into food coatings [35,40]. The ability of FBPs to provide a reservoir of antifungal compounds supports a broader trend in food packaging research: the use of agro-industrial residues for developing functional and sustainable active materials. The phenolic profile of these extracts not only contributes to antimicrobial protection but also offers environmental and economic benefits by valorizing waste streams from which we obtain FBPs [38,41].

In the in vivo assays, coatings were generally more effective in oranges than in lemons (Figure 4). This discrepancy may be attributed to a higher initial contamination level in the lemon batch, or to the possibility that the *P. digitatum* strain used for lemon inoculation was more aggressive than the one applied to oranges. Such variability in pathogen virulence and fruit susceptibility is well-documented in postharvest pathology. Additionally, the thicker and more porous peel of Verna lemons may have influenced coating adherence or compound diffusion, potentially diminishing protection effectiveness compared to oranges. Peels rich in pectin and hemicelluloses tend to be more absorbent and variable in surface integrity, which can lead to uneven coating performance and microbial infiltration [38,41].

Among the tested treatments, the grapefruit EO-enriched wax (G-EO) was particularly effective in lemons, where it delayed the onset of visible fungal decay by approximately 2 d compared to CTRL. This effect may be attributed to the synergistic interaction between carnauba wax—a hydrophobic compound known for reducing water loss and gas exchange—and the bioactivity of monoterpenes such as limonene and α-terpineol, dominant in grapefruit EO [3,6]. In oranges, the best performance was observed with the coating enriched in avocado peel extract (A-BP), which reduced disease incidence by approximately 30% compared to uncoated fruit after 10 d at 20 °C.

The strong performance of this FBP extract is particularly noteworthy, as it confirms that solid plant-based residues, when properly dried, ground, and incorporated, can serve as efficient antifungal agents in vivo. Recent studies support this observation in avocado peel BPs [42,43,44]. Also, Chen et al. [35] and Kumar et al. [38] reported that phenolic-rich plant extracts, including those from avocado peel and pomegranate rind, improved postharvest disease resistance and oxidative stability in citrus and other fruits. Furthermore, FBPs have minimal odor impact, making them more consumer-acceptable than certain EOs like clove or cinnamon, which, while highly active, may impart strong aromas that compromise fruit flavor [25,31,38].

In terms of fruit quality, the application of bioactive coatings significantly reduced weight loss over storage (Figure 5), a result attributable to the semi-permeable nature of the wax matrix. This barrier function reduces transpiration and metabolic activity, mechanisms long recognized in the postharvest field [2,6]. Interestingly, firmness was not significantly affected during the 15 d period, likely due to the relatively short storage time. Still, the coating’s ability to reduce desiccation and decay without altering texture indicates strong potential for short- to medium-term citrus preservation. In this sense, our obtained values for weight losses over the shelf-life study can be comparable to those reported by Badiche-El et al. [22] (with a maximum of 4.5–6%) in the same variety of lemons after 28 d cold storage at 2 and 10 °C. The same behavior was observed in Navelate oranges after 28 d refrigerated storage + 5 d at 20 °C [23,24], being comparable to the obtained results in the present experiment.

Overall, the in vivo results affirm that coatings enriched with natural biocompounds can act as a dual-function strategy: providing antifungal protection while maintaining critical quality attributes such as dehydration losses or firmness, in long periods of storage. When chosen and formulated appropriately, both EOs and FBPs serve as sustainable alternatives to synthetic fungicides, with avocado peel extract standing out as a particularly promising candidate for future postharvest applications.

Therefore, the in vitro assays confirm that both essential oils and fruit by-product extracts have significant potential as natural antifungal agents. While EOs such as clove and oregano offer high potency, their strong aroma poses challenges for practical application. On the other hand, phenolic-rich FBPs, particularly avocado peel, emerge as promising alternatives due to their effectiveness, mild sensory impact, and sustainability. Future work should explore the stabilization of these bioactives through encapsulation or controlled-release systems to improve their application in coatings.

In this line, other limitations of this study should be acknowledged. The storage conditions used were limited to 10 days at ~20 °C, which does not reflect commercial cold-chain environments with extended durations and fluctuating humidity. Additionally, the strong aroma of certain essential oils, such as clove and cinnamon, may negatively impact fruit flavor and consumer acceptance, underscoring the need for odor-masking or encapsulation strategies. While avocado peel-based extracts showed good potential, the scalability of coating applications and variability among citrus varieties remain unresolved. Lastly, the lack of biochemical analyses limits our understanding of the coatings’ mode of action at the molecular level.

This study demonstrates that carnauba wax-based coatings enriched with phenolic-rich natural extracts, particularly avocado peel FBP, can significantly slow down *P. digitatum* infection in organic oranges while preserving fruit quality. Based on the results obtained, several future research directions are recommended to support the commercial development of bioactive coatings. For instance, for industrial applications, it will be needed to study the thickness, adherence, release kinetics of bioactive compounds, and penetration depth studies to know the behavior of the wax on the citrus surface. Extended storage trials under real-world cold-chain conditions (e.g., 5 °C for 30–60 days) are essential to validate efficacy beyond short-term ambient storage. Encapsulation techniques such as nanoemulsions or emulsions could improve the controlled release of essential oils while minimizing odor impact. Formulating multi-component coatings by combining phenolic-rich fruit by-products with GRAS additives may enhance antifungal performance and barrier properties. Biochemical and molecular studies should explore how coatings influence the fruit’s defense responses, including antioxidant enzyme activity and gene expression. Additionally, sensory evaluation and food safety assessments will be necessary to quantify aroma impact and to ensure consumer acceptance and regulatory compliance. Finally, economic and environmental analyses, including life cycle assessments, will help determine the sustainability and scalability of these coatings as viable alternatives to synthetic postharvest fungicides.

## 5. Conclusions

This study proposes a novel and practical system by integrating EOs and FBPs into a carnauba wax matrix, offering a multifunctional edible coating with antimicrobial and antioxidant properties. This approach represents a heuristic solution for postharvest preservation, combining natural, sustainable ingredients in a lipid-based carrier with proven efficacy. Compared to the CTRL oranges, the optimized coating with avocado peels (A-BP) reduced the decay by up to ~20–25%. Furthermore, all the coated treatments reduced weight losses by ~15–25% in comparison with uncoated fruits. The use of FBPs adds value to food waste streams, aligning with circular economy principles, while carnauba wax ensures high adherence and protective properties.

Future studies should explore coating performance in larger-scale trials, evaluate consumer acceptability, and assess compatibility with other preservation strategies. Incorporating nanostructured carriers or emulsification techniques may enhance the stability and release of active compounds, while expanding to other fruit types could broaden the applicability of this approach. Ultimately, integrating sustainability, food safety, and shelf-life preservation through natural and circular solutions represents a promising direction for postharvest innovation.

## Figures and Tables

**Table 1:** Initial characterization of studied citrus fruits.

	Lemon		Orange	
Weight (g)	167.4 ± 30.3	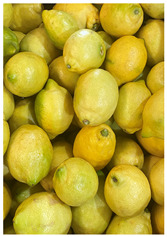	314.3 ± 26.7	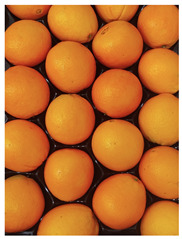
Polar diameter (mm)	97.2 ± 14.1		81.2 ± 2.8	
Equatorial diameter (mm)	63.4 ± 4.5		80.8 ± 3.2	
Peel thickness (mm)	7.9 ± 0.9		5.1 ± 0.3	
L*	69.4 ± 3.0		61.3 ± 1.7	
a*	−5.0 ± 4.2		23.7 ± 4.7	
b*	46.2 ± 3.5		55.9 ± 3.1	
Firmness (N)	17.1 ± 0.5		15.9 ± 0.4	
Juice yield (%)	28.3 ± 2.1		34.6 ± 4.6	
pH	2.4 ± 0.0		4.1 ± 0.0	
Total soluble solids (%)	8.1 ± 0.1		11.9 ± 0.8	

## Data Availability

The original contributions presented in this study are included in the article/Supplementary Materials. Further inquiries can be directed to the corresponding author.

## Supplementary Materials

The following supporting information can be downloaded at: https://www.mdpi.com/article/10.3390/foods14152616/s1, Figure S1: decay (%) and sporulation (%) percentage of inoculated organic lemon and oranges with *P. digitatum* and coated with waxes enriched in EOs (lemons) and FBPs (oranges). CTRL: control unwaxed; CTRL-W: control waxed; OR-EO: wax enriched in 0.5% oregano EO; CL-EO: wax enriched in 0.5% clove EO; G-EO: wax enriched in 0.5% grapefruit EO; A-BP: wax enriched in 5% avocado FBP; O-BP: wax enriched in 5% orange FBP; G-BP: wax enriched in 5% grapefruit FBP. *, **, and *** denotes significant results *p* < 0.05, *p* < 0.005, and *p* < 0.001, respectively, compared to CTRL.
