# Supplementary material for: Carnauba Wax Coatings Enriched with Essential Oils or Fruit By-Products Reduce Decay and Preserve Postharvest Quality in Organic Citrus
Source: Foods. 2025 Jul 25;14(15):2616. doi: 10.3390/foods14152616 (PMC12346833; doi:10.3390/foods14152616)
Supplement: Supplementary file 1 [file foods-14-02616-s001.zip › foods-3780268-supplementary.pdf]

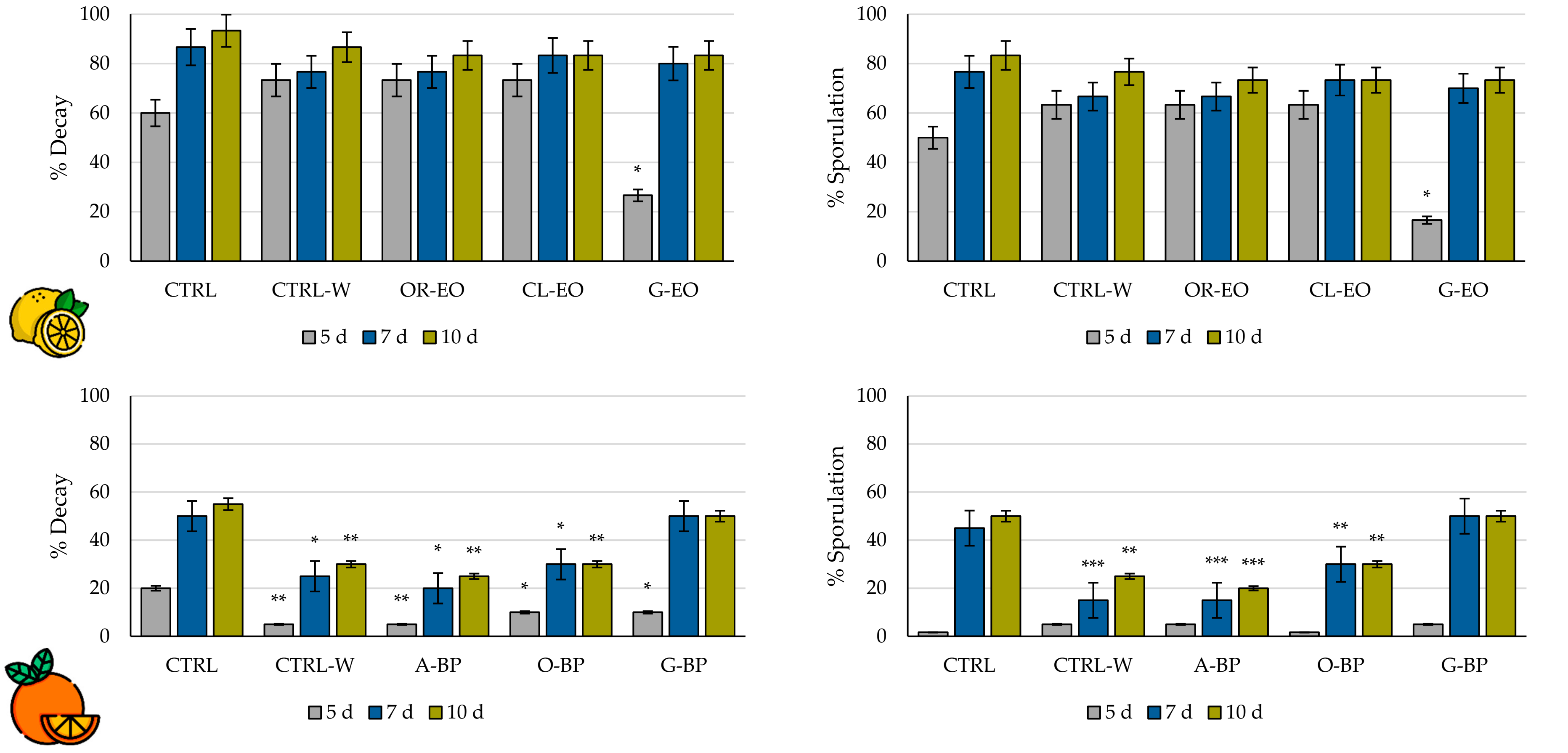

Figure S1. Decay (%) and sporulation (%) percentage of inoculated organic lemon and oranges with *P. digitatum* and coated with waxes enriched in EOs (lemons) and FBPs (oranges). CTRL: Control unwaxed; CTRL-W: Control waxed; OR-EO: Wax enriched in 0.5% oregano EO; CL-EO: Wax enriched in 0.5% clove EO; G-EO: Wax enriched in 0.5% grapefruit EO; A-BP: Wax enriched in 5% avocado FBP; O-BP: Wax enriched in 5% orange FBP; G-BP: Wax enriched in 5% grapefruit FBP. *, **, and *** denotes significant results p<0.05, p<0.005, and p<0.001, respectively, compared to CTRL.
